# Cytochrome 4Z1 Expression Is Correlated with Poor Prognosis in Patients with Cervical Cancer

**DOI:** 10.3390/curroncol28050306

**Published:** 2021-09-16

**Authors:** Yousef M. Al-saraireh, Fatemah O. F. O. Alshammari, Ahmed M. M. Youssef, Yahya M. Al-sarayra, Renata A. Al-saraireh, Ghadeer H. Al-muhaisen, Yanal S. Al-mahdy, Ahlam M. Al-Kharabsheh, Seham M. Abufraijeh, Hamzeh Mohammad Alrawashdeh

**Affiliations:** 1 Department of Pharmacology, Faculty of Medicine, Mutah University, P.O. Box 7, Al-Karak 61710, Jordan; 2 Department of Medical Lab Technology, Faculty of Health Sciences, The Public Authority for Applied Education and Training, Shuwaikh 15432, Kuwait; fo.alowayed@paaet.edu.kw; 3 Department of Pharmacology, Faculty of Pharmacy, Mutah University, P.O. Box 7, Al-Karak 61710, Jordan; Ammyouss@mutah.edu.jo; 4 Al-karak Governmental Hospital, Ministry of Health, P.O. Box 86, Al-Karak 11118, Jordan; yahyasar55@gmail.com (Y.M.A.-s.); Renatasar@yahoo.com (R.A.A.-s.); 5 Department of Microbiology and Pathology, Faculty of Medicine, Mutah University, P.O. Box 7, Al-Karak 61710, Jordan; ghadeerhm@mutah.edu.jo; 6 Department of Clinical Sciences, Faculty of Medicine, Mutah University, P.O. Box 7, Al-Karak 61710, Jordan; 120171501052@mutah.edu.jo; 7 Department of Obstetrics and Gynecology, Faculty of Medicine, Mutah University, P.O. Box 7, Al-Karak 61710, Jordan; alkharabsheh@mutah.edu.jo (A.M.A.-K.); sehammahmoud@mutah.edu.jo (S.M.A.); 8 Department of Ophthalmology, Sharif Eye Centers, Irbid 410739, Jordan; dr_hmsr@yahoo.com

**Keywords:** cancer, cervical cancer, cytochrome P450, cytochrome4z1, immunohistochemistry

## Abstract

Background: cervical cancer is one of the most common malignancies in women worldwide and its management remains challenging and complex. As Cytochrome4Z1 (CYP4Z1) is overexpressed in many tumours, its expression in cervical cancer is unknown. Therefore, the present study aimed to evaluate CYP4Z1 expression in cervical cancers. Methods: CYP4Z1 expression was immunohistochemically assessed in 100 cases of cervical cancers along with ten normal cervix tissues, and the enzyme’s relationship to several clinicopathological features and survival was explored. Results: CYP4Z1 was strongly expressed in 55% of cervical cancer patients. Normal cervix samples were negative for CYP4Z1 expression. Importantly, this expression was significantly found in patients with the late stage of the disease, lymph node metastasis, and high tumour invasion (*p* < 0.05). Interestingly, CYP4Z1 expression was significantly correlated with shorter survival times of cervical cancer patients. Univariate analysis showed that CYP4Z1 expression, tumour stage, lymph node metastasis, and tumour invasion were significantly correlated with patient survival (*p* < 0.05). The multivariate analysis revealed that only CYP4Z1 expression and tumour stage were significantly correlated with patient survival (*p* < 0.05). Conclusions: CYP4Z1 expression is associated with cervical cancer patients’ survival and may serve as an independent predictor of poor prognosis in cervical cancer patients.

## 1. Introduction

Cervical cancer ranks as the fourth most prevalent cause of cancer mortality and morbidity in women worldwide [1,2]. According to the International Agency for Research on Cancer report GLOBOCAN 2018, the annual cervical cancer burden reaches 570,000 new cases and about 311,000 deaths from cervical cancers globally [3]. This type of tumour is mainly triggered and developed by persistent infection by human papilloma virus (HPV). This is a sexually transmitted virus that is classified into high-risk and low-risk types. In particular, the most common aggressive types of virus, causing approximately 70% of cervical cancers, are HPV 16 and 18 [4,5]. In recent decades, the burden of HPV in cervical cancer has decreased because of the effective implementation of cervical screening and HPV vaccination programmes and improvements in therapeutic strategies. However, cervical cancer mortality remains high in some regions of the world, particularly in developing countries, due to a lack of screening and vaccination programmes [2,5,6]. Based on these facts, there should be an urgency to accelerate the development of novel biomarkers and targeted therapies to better manage cervical cancer.

The role and significance of cytochrome P450s (CYPs) in the carcinogenic process have contributed to the development of cancer therapies based on the expression and metabolic pathways of CYPs [7]. Of specific interest is the aberrant expression of CYP4Z1 in breast cancer. CYP4Z1 selective expression in breast cancer has inspired researchers to characterise its expression in other cancer types and question its effect on cancer development [8,9,10,11]. Clinical studies exploring the expression profile of orphan CYP4Z1 and its association with clinicopathological parameters, albeit limited, demonstrate an interesting trend. Several studies reported differential expression of CYP4Z1 in cancers of the breast, ovary, and prostate [8,11,12,13]. Recently, we characterised CYP4Z1 expression in bladder [14]. Importantly, its expression is associated with poor patient prognosis and has been suggested as a biomarker for cancers of the ovary and prostate [12,13]. Moreover, the expression of CYP4Z1 was able to differentiate between benign, primary, and malignant breast and ovarian tumours [15]. Of interest is the finding that the cell surface of breast cancer has shown an abnormal translocation of CYP4Z1 expression compared to nothing displayed on the surface of normal breast cells [16]. This aberrant cell surface localisation enhances the development of CYP4Z1 autoantibodies in breast cancer patients’ sera and is proposed as a diagnostic biomarker for breast cancer [17]. Therefore, CYP4Z1 may show potential as a biomarker and in the development of targeted therapies selectively directed at the tissues in which it is expressed.

The high expression and poor prognosis association of CYP4Z1 prompted both in vitro and in vivo studies to unravel the enzyme’s contribution to tumour development. CYP4Z1 was conditionally overexpressed in breast cancer cells when treated with glucocorticoids and progesterone. Interestingly, treatment of these breast cancer cells with a steroid-receptor blocker, mifepristone, reduced CYP4Z1 conditional overexpression [18]. Importantly, CYP4Z1 expression significantly enhanced tumour growth, angiogenesis, and spread of cancer cells in both in vitro and in vivo models. These effects were biochemically accompanied by a reduction in fatty-acid levels, particularly lauric and myristic acids, and an increase in 20-hydroxyeicosatetraenoic acid (20-HETE) levels [19]. Such biochemical hydroxylase and epoxygenase activities of CYP4Z1 were reported by several studies [20,21,22,23]. CYP4Z1 was capable of metabolising lauric and myristic acids to monohydroxylated products and arachidonic acid to 20-HETE [21]. However, a recent report has identified CYP4Z1 to exclusively metabolise arachidonic acid to14,15-epoxyeicosatrienoic acid (14,15-EET) rather than 20-HETE [22]. Overall, CYP4Z1 biochemical activities towards arachidonic acid metabolism to either 20-HETE or 14,15-EET have been proposed as a causative mechanism contributing to tumour development [22,23]. This may provide a possible molecular pathway for CYP4Z1-driven tumour progression. However, the CYP4Z1 enzyme’s association with cancer development remains the focus of current research.

In a recent pilot study, we detected CYP4Z1 as being strongly expressed in a small number of cervical cancers [11]. Human protein atlas results on CYP4Z1 mRNA profiling in 291 cervical cancer patients demonstrated that 93 (28.5%) patients had elevated expression, while the remaining 208 patients (71.5%) showed low expression [24]. Therefore, there remains a question about CYP4Z1 protein expression profile in cervical cancers relative to normal cervix tissues. On this basis, our observations were expanded to investigate aberrant CYP4Z1 expression in a large number of tissues including cervical cancers and the normal cervix, and to explore its relation to demographic and clinicopathologic features as well as patient survival.

## 2. Materials and Methods

### 2.1. Tissue Specimens

Prior to conducting the study, patient consent was waived due to an exemption for the use of archived wax cervical tissue samples issued by the Institutional Review and Ethics Committee, Faculty of Medicine, University of Mutah (Reference No. 4012021 date: 20 January 2021). The study was carried out according to ethical guidelines set out by the Declaration of Helsinki (2013). Clinical human tissues of cervical cancer and the normal cervix were submitted to the pathology department of the King Hussein Medical Hospital, Royal Medical Services, Amman and the King Abdullah University Hospital, Irbed, Jordan. Tissues were fixed in 10% neutral buffered formalin, and paraffin tissue blocks were prepared through the wax-embedding process. Tissue sections, 5 µm thick, were performed and stained with haematoxylin and eosin for routine diagnosis. Tissue samples for cervical cancer and the normal cervix were available from 100 patients. Of these samples, 95 were squamous cell carcinomas, while the remaining samples were three adenocarcinomas and two endometrioid adenocarcinomas. Ten tissue samples were available from patients with normal pathology of the cervix. None of the patients in this study had had chemotherapy or radiotherapy. All available patient data regarding age, tumour histopathology, tumour grade, clinical stage, and expression of HPV16/18 and Ki67 were extracted from the patients’ files. For expression of HPV16/18, tissue specimens were scored according to the following scale: negative (0), low (1), moderate (2), and high (3). A score of 0 (negative) was applied to a lack of expression or cells only showing staining less than 5%. A score of 1 (low) corresponded to cells showing immunoreactivity from 5% to 33%. Tissue sections showing immunoreactivity between 33% and 66% of the cells were allocated 2 (moderate). A score of 3 (high) was applied to tissue sections showing immunoreactivity over 67% of the cells. Regarding the Ki67, a score of low, moderate, or high was allocated when the Ki-67 expression was less than 5%, 5–30%, and more than 31%, respectively. As the major pathological subtype in this study was squamous cell carcinoma (95 cases), the survival data for these patients were retrieved from follow-up records ranging from 3 to 60 months (median, 49 months). Overall survival was calculated from the date of surgery to the date of death or the date of last follow-up visit. The personal details of the patients were kept private and anonymous.

### 2.2. Immunohistochemistry

The tissue sections were dewaxed in xylene and rehydrated through successive treatment with serial dilutions of alcohol. Sections were then treated with 3% hydrogen peroxide to inhibit endogenous peroxidase activity. Following a wash with PBS, tissues were microwaved in a citrate buffer for 20 min to retrieve the antigen. To eliminate non-specific-antibody binding, tissue sections were treated with 2.5% normal goat serum. After that, tissue samples were incubated with rabbit polyclonal antibody specific for CYP4Z1 at concentration of 5 μg/mL overnight at 4 °C (NBP1-91817, Novus biological, Englewood, CO, USA). Using western blotting, the specificity of the given antibody towards CYP4Z1 was confirmed using whole CYP4Z1 isogenic cell lysates. After being washed, ImmPRESS (Peroxidase) Polymer Goat Anti-Rabbit IgG Reagent was applied to each tissue section for 30 min (MP-7451, Vector Laboratories, Burlingame, CA, USA). Following a wash, colour development was performed by incubating the tissue sections with 3,39-diaminobenzidine chromogen substrate (DAB). Sections were then counterstained with haematoxylin, dehydrated through successive treatment with serial dilutions of alcohol to xylene, and mounted with coverslips. The positive control for immunoreactivity was breast cancer tissue. The negative controls was tissue incubated with normal goat serum instead of the primary antibody. Additionally, the CYP4Z1 antibody specificity was further validated by pre-incubating the CYP4Z1 antibody with CYP4Z1 blocking peptide (H00199974-P01, Novus biological, Englewood, CO, USA) at room temperature for one hour. The resulting combination was then used instead of the CYP4Z1 primary antibody to block subsequent primary-antibody binding to the epitope of CYP4Z1 in specimen. The degree of staining was evaluated between samples treated with the CYP4Z1 antibody and samples treated with the blocked antibody. The slides were evaluated using a Leica DMRB microscope equipped with a JVC video camera (Leica DMRB, Wetzlar, Germany) and images were digitally processed using AcQuis imaging capture bio-software system (Synoptics, Cambridge, UK).

### 2.3. Scoring

Two independent pathologists semi-quantitatively assessed the results of immunohistochemistry. The expression of CYP4Z1 was considered positive when the membrane and/or cytoplasm of cells showed yellow or brown colour. Tissue specimens were rated for the density and degree of CYP4Z1 expression based on the following scale: negative (0), low (1), moderate (2), and high (3). A score of 0 (negative) was applied to a lack of expression or cells only showing staining less than 5%. A score of 1 (low) corresponded to cells showing immunoreactivity from 5% to 33%. Tissue sections showing immunoreactivity between 33% and 66% of the cells were allocated 2 (moderate). A score of 3 (high) was applied to tissue sections showing immunoreactivity over 67% of the cells.

### 2.4. Statistical Analysis

Data analysis was performed using SPSS version-19 (Statistical Packages for Social Sciences, version 19). All variables were expressed in simple measures of frequency and percentage. The differences between the discrete variables were measured by using Pearson’s Chi-square test and the one-way analysis of variance (ANOVA) test when applicable. The Kaplan–Meier method was used to calculate the overall survival of patients and statistical significance was assessed by log–rank test. Univariate and multivariate Cox regression were used to identify prognostic variables correlated with patient survival. *p* < 0.05 was considered significantly different.

## 3. Results

### 3.1. Baseline Demographic and Clinicopathologic Features

This study comprised 100 females with cervical cancer and a control group of ten females with normal cervix pathology, with an average age of 50 ± 10.2 (Table 1). Of the patients, 62.7% (69 cases) were under 50 years of age, while 37.3% (41 cases) were over 50 years of age. In this study, the most popular pathology of cervical cancer was squamous cell carcinoma (95, 95%). Other cervical cancer types included three (3%) adenocarcinomas and two (2%) endometrioid adenocarcinomas. Just over half of the patients were at tumour stage I (56%, 56 cases), while 38% (38 cases) and 6% (six cases) of patients were at stage II and stage III, respectively. Regarding the tumour grade, the majority of the patients were at grade III (64%, 64 cases) and grade II (27%, 27 cases), whereas not many constituted grade I (9%, nine cases). Moreover, slightly over half of the patients (59%, 59 cases) had tumours confined to the uterus (T1), and one-third of the patients (38%, 38 cases) had tumour invasion beyond the uterus but not to the pelvic wall or vagina (T2). Patients with tumour invasion to the pelvic wall or vagina (T3) were 3% (three cases). For statistical analysis purposes, lymph node metastasis status was either graded as lymph node-positive (95%, 95 cases) or lymph node-negative (5%, five cases). Additionally, the pathology assessment indicated that all patients included in this study were free from distant metastasis. Furthermore, 46% (46 cases) of patients had moderate HPV16/18 expression, while 22% (22 cases) and 32% (32 cases) of patients had low and high HPV16/18 expression, respectively. For Ki67 expression, almost half of patients had low expression (47%, 47 cases), while other patients demonstrated moderate (35%, 35 cases) and high (18%, 18 cases) expression.

### 3.2. Prevalence of CYP4Z1 Expression

**Figure 1:**
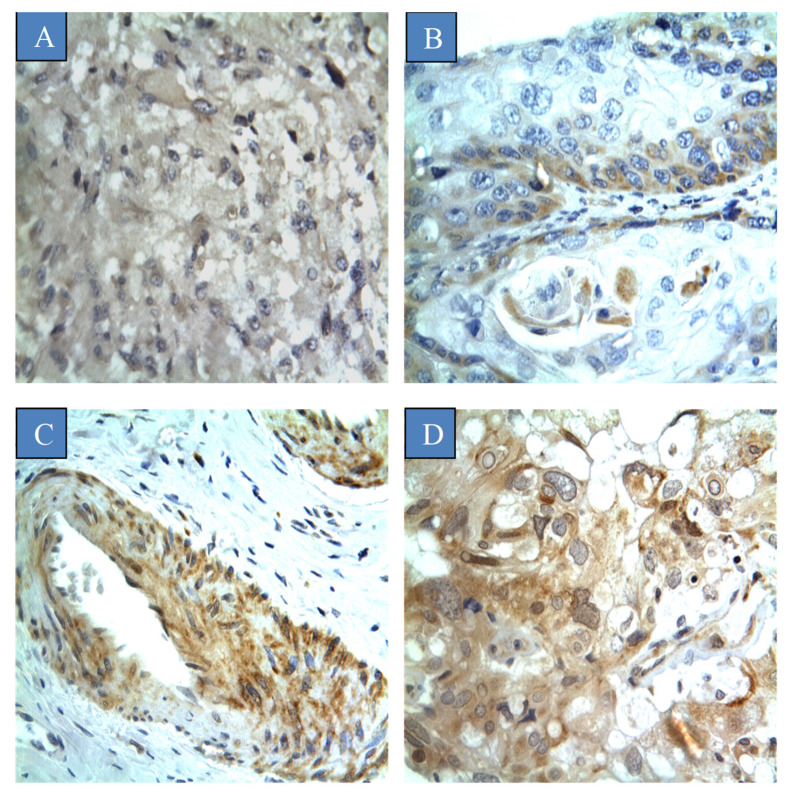
Different scores of CYP4Z1 expression in cervical cancer tissues: (**A**) score ‘negative’ showing no expression in the tissue at all; (**B**) score ‘low’ showing expression less than 33% of cells; (**C**) score ‘moderate’ showing expression in 34–66% of the cells, and; (**D**) score ‘strong’ showing expression in more than 67% of the cells. Magnification (×400).

**Figure 2:**
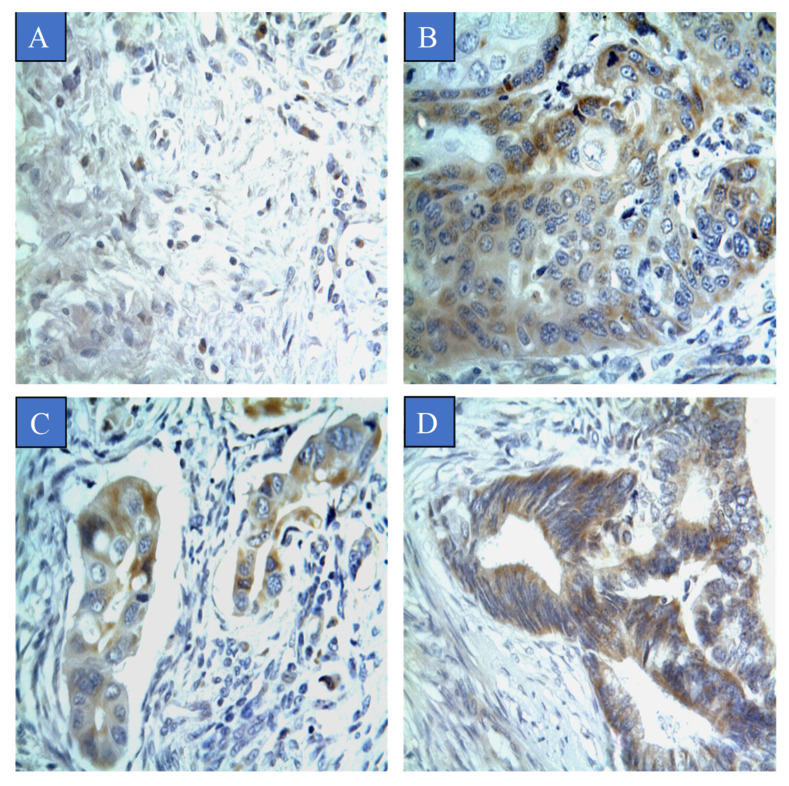
CYP4Z1 expression in different types of cervical cancer. Tumours were classified on the basis of pathological subtype: (**A**) normal cervix tissue; (**B**) squamous cell carcinoma; (**C**) adenocarcinoma, and; (**D**) endometrioid adenocarcinoma. Magnification (×400).

The scoring criteria for semi-quantitative assessment of CYP4Z1 expression is shown in Figure 1. CYP4Z1 expression was identified in 55% (55 cases) of cervical cancers. The expression was primarily localised to the surface or cytoplasm of cells without substantial nucleus staining. There was no CYP4Z1 expression displayed in normal cervix tissues (Figure 2). Importantly, CYP4Z1 expression was validated by using proper positive and negative controls and by inhibition of immunoreactivity using CYP4Z1-blocking peptide. High CYP4Z1 expression was displayed in the positive control breast cancer tissue, while no expression was observed in the negative control. Weak to no CYP4Z1 expression was exhibited in cervical and breast cancer tissues treated with a mixture of CYP4Z1 primary antibody and blocking peptide (Figure S1). 

The association between CYP4Z1 expression and various demographic and clinicopathologic characteristics was examined (Table 1). CYP4Z1 expression was found to be significantly associated with tumour pathological subtype, tumour stage, tumour invasion, and lymph node metastasis (*p* < 0.05). There was a significant differential in CYP4Z1 expression between normal cervix tissues and various cervical pathological subtypes (*p* < 0.05). Of positive patients, CYP4Z1 was expressed in 50% (50 cases) of patients with squamous cell carcinoma. All patients with adenocarcinoma and endometrioid adenocarcinoma exhibited CYP4Z1 expression (Figure 2). Regarding the histological stage, CYP4Z1 was more frequently expressed in patients with stage II (97.4%, 37 cases) and stage III (100%, six cases) than in stage I (16.1%, 9 cases). Additionally, a high rate of CYP4Z1 expression was found in patients having tumour depth of invasion of T3 (100%, three) and T2 (97.4%, 37 cases), but not T1 (20.4%, 15 cases). Moreover, all the lymph node metastatic patients had high CYP4Z1 expression. Additionally, there were no significant associations found between CYP4Z1 expression and age of patients, tumour grade, and expression status of HPV16/18 and Ki67. 

### 3.3. Survival Analysis and Prognostic Value of CYP4Z1 Expression

**Figure 3:**
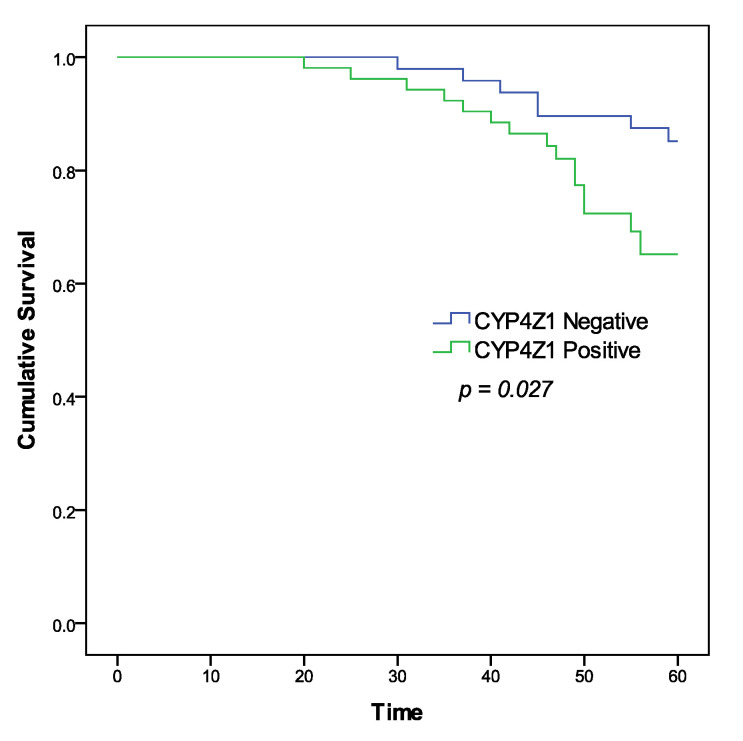
Kaplan–Meier survival curve of cervical cancer patients according to CYP4Z1 expression.

As the squamous cell carcinoma was the major pathological subtype in this study (95 cases), the overall survival and prognostic utility of CYP4Z1 expression in squamous cell carcinomas were analysed. For statistical purposes, patients were grouped as patients with negative CYP4Z1 expression (45%, 45 cases) and patients with positive CYP4Z1 expression (50%, 50 cases). Patient survival data were analysed using the Kaplan–Meier curve and the log–rank test in relation to patient groups. Results showed a significant correlation of CYP4Z1 expression with cervical patient survival (*p* = 0.027) (Figure 3). Patients with CYP4Z1 expression had a poor survival rate (78%), compared to patients with negative CYP4Z1 expression (86.7%). In univariate analysis, CYP4Z1 expression, histological stage, tumour invasion, and lymph node metastasis were significantly correlated with overall survival, with *p*-values of 0.002, 0.007, 0.008 and 0.003, respectively. In multivariate analysis, CYP4Z1 expression was found to be an independent prognostic predictor of poor cervical cancer patient survival (*p* = 0.034; HR 1.113, 95% CI = 1.059–1.743), along with another prognostic factor, histological stage (*p* = 0.023; HR 7.384, 95% CI = 3.318–11.381) (Table 2).

## 4. Discussion

Cervical cancer has become a major health concern due to the rise in mortality and morbidity around the world [2,3]. This rise, particularly in developing countries, is attributed to poor screening and vaccination programmes against HPV [3,5,6]. This disease is considered more aggressive and has a relatively worse prognosis [3,4]. Owing to the lack of targeted therapies, the clinical management of cervical cancer is challenging and remains difficult. As a result, there is an important need to find novel biomarkers and drug targets that can improve cervical cancer clinical management. While considered an interesting area of study, new and existing research opportunities emerge to unveil novel aspects of CYP4Z1 in cancer development and therapy. Our recent initial screening has identified overexpression of CYP4Z1 in a small number of tumour samples for each type of human tumour including cervical cancer [11]. Therefore, the implications of this observation have been taken to fully characterise the CYP4Z1 expression in a large cohort of cervical cancers.

As the current study was the first investigating the CYP4Z1 expression in cervical cancers, we found that 55% of the tumours expressed CYP4Z1, where the expression in each tumour sample was specifically confined to tumour cells. Normal cervix tissues showed no CYP4Z1 expression at all. The high frequency of CYP4Z1 expression in cervical cancer is consistent with the findings of our earlier initial screening [11]. Moreover, our results agree with CYP4Z1 transcription profiling in cervical cancers shown by the Human Protein Atlas. Low to high CYP4Z1 mRNA levels were identified in cervical cancers compared to normal cervix tissues [24]. Importantly, this CYP4Z1 differential expression between normal tissues and tumour tissues was significantly observed in many studies. In these studies, CYP4Z1 was expressed at much higher levels in cancers of the breast, ovary, and prostate than in their corresponding normal tissues [8,10,11]. Moreover, we recently identified a similar fashion of CYP4Z1 differential expression in bladder [14] and colon cancers (data not published). 

The role of the CYP4Z1 enzyme as a prognostic marker in cervical cancer was assessed in this study. This was the first study indicating a significant correlation between CYP4Z1 expression and cervical cancer patients’ survival. CYP4Z1 expression was associated with poor survival rate and identified as an independent factor for poor prognosis in cervical cancer patients, along with tumour stage. These findings are in agreement with previous studies identifying CYP4Z1 as a prognostic marker for ovarian and prostate cancers [12,13]. Further significant associations were found between CYP4Z1 expression and tumour invasion and lymph node metastasis. These findings demonstrate the possible role of the CYP4Z1 enzyme in the progression and malignancy of cervical cancer.

As few functional studies have interrogated the role of CYP4Z1 in cancer development particularly breast cancer [16,19,20,25], mechanisms behind functions of CYP4Z1 in cervical cancer progression are still unknown. By using in vitro and in vivo models, CYP4Z1 overexpression was found to promote breast cancer-cell invasion, migration, proliferation, and tumour angiogenesis [16,19,20]. This was particularly triggered by activation of ERK1/2 and PI3K/Akt signalling pathways through increased expression of vascular endothelial growth factor-A (VEGF-A) and decreased production of the tissue inhibitor of metalloproteinase-2. It is important to note that all of these changes were biochemically associated with increased production of 20-HETE and 14,15-EET [19,22]. Such CYP4Z1 enzymatic activity of metabolising arachidonic acid to either 20-HETE or 14,15-EET was reported by many studies [21,22]. Importantly, the 20-HETE was shown in many studies to work in conjugation with VEGF, enhancing tumour angiogenesis, growth, and metastasis [26,27]. Beside VEGF, 20-HETE was shown to be involved in activation of the PI3K/Akt- and mitogen-activated protein kinase (MAPK) pathways necessary for proliferation and survival of cancer cells [28]. Regarding 14,15-EET, it was reported that 14,15-EET promoted tumour angiogenesis by stimulating tyrosine-protein kinase (Src) and activated transcription-3 (STAT-3)-dependent production of VEGF [29]. Moreover, 14,15-EET was found to partly regulate the pro-tumourigenic pathways of PI3K/Akt, MAPK, and VEGF [22,30,31]. The activation of these signalling pathways in HPV-induced cervical cancers wwas reported by numerous studies [32,33,34]. Further analysis of the mechanistic role of CYP4Z1 in the tumourigenesis process showed that synergic expression of pseudogene CYP4Z2P and CYP4Z1-3’UTRs enhanced tumour neovascularization in breast cancer partly through activating pathways of PI3K/Akt and ERK1/2 [20]. Moreover, overexpression of CYP4Z1 and/or CYP4Z2P in breast cancer cells may promote transcriptional activity of oestrogen receptors, stemness, and tamoxifen resistance [25]. Taken as a whole, these findings may provide a plausible mechanism for CYP4Z1-driven tumour development. However, the link between the CYP4Z1 enzyme and cervical cancer development remains elusive.

Knowledge in the field of the CYP4Z1 enzyme’s substrate recognition and catalytic properties is now quite valuable in the design and development of more selective cancer therapies. A limited number of reports explored the substrate-binding mode of CYP4Z1 [35,36,37,38]. Several key amino acid residues have been identified for substrate binding of CYP4Z1 including Arg487, Asn381, Ser383, Ser222, Ser113, and Asn381 [35,36]. Recently, luciferin benzyl ether was identified as the best luminogenic substrate for CYP4Z1 using the permeabilised cells of fission yeast expressing CYP4Z1 [36]. These recent advances in determining the CYP4Z1 enzyme’s substrate recognition have led to the development of selective inhibitors for CYP4Z1. The first inhibitor identified was 1-benzylimidazole, which showed efficient blocking ability for production of 14, 15-EET in CYP4Z1 positive tumour cells relative to a poor inhibitory profile against other CYPs [38]. Interestingly, a new highly potent inhibitor (Compound 9) for CYP4Z1 was developed using systematic virtual screening. This novel inhibitor showed selective binding and high nanomolar affinity to CYP4Z1 [37]. These latest advances may accelerate the development of CYP4Z1 targeted therapies.

The future of cancer therapy relies mainly on the use of biomarkers that guide clinicians in each step of cancer management. For this purpose, there is an increasing interest in discovery and development of novel biomarkers that help in cancer diagnosis, prognosis, and monitoring treatment response [39]. For cancer diagnosis, biomarkers can be used for cancer risk assessment, early detection of cancer, and accurate staging of disease. For instance, analysis of differentially expressed protein in discrimination between normal and cancer tissues [40]. Regarding cancer prognosis, biomarkers help in estimating the course of cancer disease and therefore the most suitable management strategy. Moreover, biomarkers can also be used for predicting the response of patients to various treatment strategies [41]. In this case, for example, the biomarker expression levels generally reflected the extent of cancer burden, therefore, high levels of biomarker mostly indicated poor prognosis and sometimes the opposite. As it reflected the cancer burden, biomarkers could also be used in the cancer staging system [40]. Overall, the discovery of novel biomarkers probably CYP4Z1 may hold promise for cervical cancer management.

## 5. Conclusions

Unique CYP4Z1 expression was identified in 55% of cervical cancers compared to negative expression in normal cervix tissues. This expression was strongly found to be at a high frequency in patients with the late stage of the disease, lymph node metastasis, and high tumour invasion. Importantly, CYP4Z1 expression was significantly correlated with a shorter survival rate and poor prognosis of cervical cancer patients. Taken together, these interesting findings may offer a new novel avenue for biomarker and therapy development in cervical cancers.

## Figures and Tables

**Table 1:** Baseline demographic and clinicopathologic features of cervical cancers.

CYP4Z1 Expression					
Characteristic	Negative	Low	Medium	High	*p* Value
Age:					
<50 (*n* = 69, 62.7%)	30 (43.5%)	12 (17.4%)	19 (27.5%)	8 (11.6%)	0.104
≥50 (*n* = 41, 37.3%)	25 (61%)	3 (7.3%)	12 (29.3%)	1 (2.4%)	
Pathology subtype:					
Squamous cell carcinoma (*n* = 95, 86.4%)	45 (47.4%)	15 (15.8%)	29 (30.5%)	6 (6.3%)	0.011
Adenocarcinoma (*n* = 3, 2.7%)	0 (0%)	0 (0%)	1 (33.3%)	2 (66.7%)	
Endometrioid adenocarcinoma (*n* = 2, 1.8%)	0 (0%)	0 (0%)	1 (50%)	1 (50%)	
Normal (*n* = 10, 9.1%)	10 (100%)	0 (0%)	0 (0%)	0 (0%)	
Histological grade:					
I (*n* = 9, 9%)	4 (44.4%)	1 (11.1%)	3 (33.3%)	1 (11.1%)	0.594
II (*n* = 27, 27%)	10 (37%)	3 (11.1%)	12 (44.4%)	2 (7.4%)	
III (*n* = 64, 64%)	34 (53.1%)	11 (17.2%)	16 (25%)	3 (4.7%)	
Histological stage:					
I (*n* = 56, 56%)	47 (83.9%)	9 (16.1%)	0 (0%)	0 (0%)	0.001
II (*n* = 38, 38%)	1 (2.6%)	6 (15.8%)	31 (81.6%)	0 (0%)	
III (*n* = 6, 6%)	0 (0%)	0 (0%)	0 (0%)	6 (100%)	
Tumour invasion:					
T1 (*n* = 59, 59%)	47 (79.7%)	9 (15.3%)	0 (0%)	3 (5.1%)	0.007
T2 (*n* = 38, 38%)	1 (2.6%)	6 (15.8%)	31 (81.6%)	0 (0%)	
T3 (*n* = 3, 3%)	0 (0%)	0 (0%)	0 (0%)	3 (100%)	
Lymph node metastasis:					
Negative (*n* = 95, 95%)	48 (50.5%)	15 (15.8%)	31 (32.6%)	1 (1.1%)	0.003
Positive (*n* = 5, 5%)	0 (0%)	0 (0%)	0 (0%)	5 (100%)	
Distant metastasis:					
Negative (*n* = 100, 100%)	48 (48%)	15 (15%)	31 (31%)	6 (6%)	0.203
Positive (*n* = 0, 0%)	0 (0%)	0 (0%)	0 (0%)	0 (0%)	
HPV16/18 status:					
Low (*n* = 22, 22%)	13 (59.1%)	1 (4.5%)	8 (36.4%)	0 (0%)	0.231
Moderate (*n* = 46, 46%)	18 (39.1%)	10 (21.7%)	13 (28.3%)	5 (10.9%)	
High (*n* = 32, 32%)	17 (53.1%)	4 (12.5%)	10 (31.3%)	1 (3.1%)	
Ki67 status:					
Low (*n* = 47, 47%)	22 (46.8%)	6 (12.8%)	16 (34%)	3 (6.4%)	0.749
Moderate (*n* = 35, 35%)	19 (54.3%)	6 (17.1%)	8 (22.9%)	2 (5.7%)	
High (*n* = 18, 18%)	7 (38.9%)	3 (16.7%)	7 (28.9%)	1 (5.6%)	

**Table 2:** Univariate and multivariate analyses of prognostic variables correlated with cervical cancer patients’ survival.

Prognostic Variable	Univariate			Multivariate		
	HR	95%Cl	*p*-Value	HR	95% Cl	*p*-Value
Age	0.977	0.930–1.127	0.364	0.997	0.943–1.054	0.911
Histological grade	1.818	0.659–5.013	0.248	1.308	0.580–2.952	0.518
Histological stage	8.422	3.486–13.347	0.007	7.384	3.318–11.381	0.023
Tumour invasion	3.445	1.382–8.589	0.008	0.363	0.267–1.621	0.084
Lymph node metastasis	7.995	4.778–12.253	0.003	6.543	3.979–11.416	0.061
HPV16/18	1.334	0.690–2.619	0.385	1.135	0.535–2.405	0.741
Ki67	0.730	0.371–1.438	0.363	0.649	0.334–1.261	0.202
CYP4Z1 expression	1.531	1.154–1.943	0.002	1.113	1.059–1.743	0.034

## Data Availability

The data presented in this study are available on request from the corresponding author. The data are not publicly available due to privacy and ethical concerns.

## Supplementary Materials

The following is available online at https://www.mdpi.com/article/10.3390/curroncol28050306/s1, Figure S1: CYP4Z1 expression in different types of experimental controls.
