# Supplementary material for: Cytochrome 4Z1 Expression Is Correlated with Poor Prognosis in Patients with Cervical Cancer
Source: Curr Oncol. 2021 Sep 16;28(5):3573–84. doi: 10.3390/curroncol28050306 (PMC8482276; doi:10.3390/curroncol28050306)
Supplement: Supplementary file 1 [file curroncol-28-00306-s001.zip › curroncol-1352391-supplementary.pdf]

Current Oncology

Supplementary Materials

Cytochrome 4Z1 Expression is Correlated with Poor Prognosis in Patients with Cervical Cancer

Yousef M. Al-saraireh, Fatemah O. F. O. Alshammari, Ahmed M. M. Youssef, Yahya M. Al-sarayra, Renata A. Al-saraireh, Ghadeer H. Al-muhaisen, Yanal S. Al-mahdy, Ahlam M. Al-Kharabsheh, Seham M. Abufraijeh and Hamzeh Mohammad Al-rawashdeh

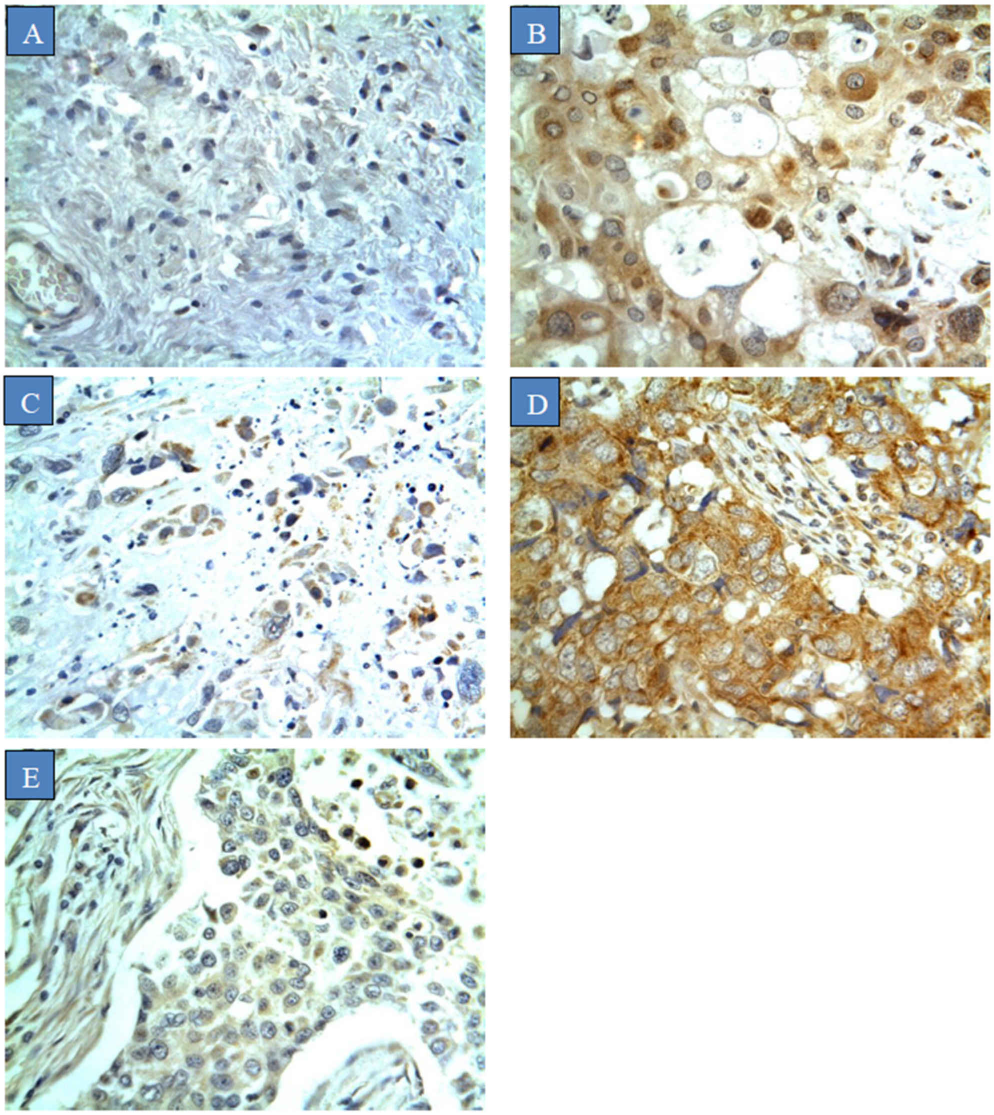

Figure S1. CYP4Z1 expression in different types of experimental controls. (**A**) No CYP4Z1expression in cervical cancer tissue incubated with normal goat serum instead of CYP4Z1primary antibody (negative control), (**B**) Elevated CYP4Z1expression was exhibited in cervical cancer tissue incubated with CYP4Z1primary antibody , (**C**) Weak to no expression of CYP4Z1 was displayed in cervical cancer tissue incubated with mixture of primary antibody and blocking peptide, (**D**) Elevated CYP4Z1expression was seen in breast cancer tissue incubated with CYP4Z1primary antibody (positive control) and (**E**) Very weak expression of CYP4Z1 was displayed in breast cancer tissue incubated with mixture of primary antibody and blocking peptide. Magnification (×400).

Citation: Al-saraireh, Y.M.; Alshammari, F.O.F.O.; Youssef, A.M.M.; AL-sarayra, Y.M.; Alsaraireh, R.A.; Al-muhaisen, G.H.; Al-mahdy, Y.S.; Al-Kharabsheh, A.M.; Abufraijeh, S.M.; Al-rawashdeh, H.M. Cytochrome 4Z1 Expression is Correlated with Poor Prognosis in Patients with Cervical Cancer. *Curr. Oncol.* **2021**, *28*, 3573–3584. https://doi.org/10.3390/curroncol28050306

Received: 6 August 2021
Accepted: 11 September 2021
Published: 16 September 2021

Publisher's Note: MDPI stays neutral with regard to jurisdictional claims in published maps and institutional affiliations.

Copyright: © 2021 by the authors. Licensee MDPI, Basel, Switzerland. This article is an open access article distributed under the terms and conditions of the Creative Commons Attribution (CC BY) license (http://creativecommons.org/licenses/by/4.0/).
